# Efficacy of Platelet-Rich Plasma in Retarding Intervertebral Disc Degeneration: A Meta-Analysis of Animal Studies

**DOI:** 10.1155/2017/7919201

**Published:** 2017-07-02

**Authors:** Pei Li, Ruijie Zhang, Qiang Zhou

**Affiliations:** ^1^Department of Orthopedic Surgery, Southwest Hospital, Third Military Medical University, Chongqing 400038, China; ^2^Department of Respiratory Medicine, The Third Xiangya Hospital, Central South University, Changsha, Hunan 410013, China

## Abstract

**Objectives:**

Several animal studies have demonstrated the positive effects of platelet-rich plasma (PRP) on disc degeneration retardation. The present meta-analysis was to verify the efficacy of PRP in retarding disc degeneration in animal.

**Methods:**

Relevant studies were identified and evaluated according to our inclusion and exclusion criteria. The standardized mean difference (SMD) and related 95% confidence interval (95% CI) were estimated to assess PRP efficiency.

**Results:**

In total, eleven studies were included in this meta-analysis. Significant differences were found in the PRP treatment group, which showed increased disc height (SMD = 2.66, 95% CI: 1.86, 3.47, *p* = 0.000), increased MRI T2 signal intensity (SMD = −3.29, 95% CI: −4.44, −2.13, *p* = 0.000), and decreased histological degeneration grade (SMD = −4.28, 95% CI: −5.26, −3.30, *p* = 0.000). However, no significant increase in collagen II expression was found (SMD = 25389.74, 95% CI: −27585.72, 78365.21, *p* = 0.348). Apart from the subgroup analysis of the disc height based on animal species (pig) and disc degeneration model (chymopapain induction), other subgroup analysis based on animal species (rabbit and rat), study design, disc degeneration model, and follow-up period demonstrated that PRP treatment can significantly restore disc height and increase MRI T2 signal intensity.

**Conclusions:**

PRP treatment is potentially effective in restoring disc height of rodent rabbit and rat, reducing histological degeneration grade, and increasing MRI T2 image signal. PRP injection may be promising therapy for retarding disc degeneration.

## 1. Introduction

Intervertebral disc degeneration (IDD) is a main contributor to low back pain (LBP) which leads to tremendous medical expenditure [1–7]. No effective treatment for IDD currently exists to reverse and repair disc degeneration. During recent decades, finding an effective treatment for and identifying the molecular biological mechanism of IDD become research priorities.

Anatomically, the intervertebral disc (IVD) consists of the central highly hydrated nucleus pulposus (NP), the peripheral lamellae annulus fibrosus (AF), and the superior and inferior cartilage endplates (CEPs) [8]. Physiologically, the swelling pressure within the NP region caused by the negatively charged proteoglycans facilitates spinal load absorption [9]. During IDD, the disc undergoes complex biochemical and cellular changes including loss of proteoglycan content, the transition of type II collagen to type I collagen, and decreases in NP cell density [10–12]. These degenerative changes directly lead to attenuated mechanical function of the IVD and ultimately induce structural disruptions, such as AF tears and NP protrusion.

IVD has a limited ability to self-repair, and the degenerative process usually progresses until it is irreversible [13]. Therefore, developing effective treatments for disc degeneration has become imperative. Previously, growth factors, such as transforming growth factor (TGF)-*β*1 [14–16], bone morphogenetic protein (BMP)-7 [17–19], growth differentiation factor-5 [20], insulin-like growth factor (IGF)-1 [17, 21, 22], basic fibroblast growth factor [23] and platelet-derived growth factor (PDGF) [23–25], were adopted to restore matrix synthesis and disc cell viability in vivo or in vitro. However, these recombinant growth factors have certain practical disadvantages, and few are available in clinical practice [26].

Platelet-rich plasma (PRP) is easily collected from the peripheral blood. It is a growth factor cocktail that contains numerous growth factors, including TGF-*β*1, IGF-1, VEGF, EGF, FGF, HGF, and PDGF [27]. Several cell culture studies have demonstrated that PRP could effectively promote disc cell proliferation and enhance extracellular matrix synthesis [28–32]. Furthermore, some in vivo animal studies have demonstrated that PRP injection into a degenerated disc could restore disc height, increase NP hydration, and upregulate the expression of matrix macromolecules [26, 29, 33–42].

Though multiple animal studies have investigated the efficacy of PRP treatment in retarding disc degeneration, single studies often have limited sample size, and the experimental designs differ across studies. Because a consensus on the efficacy of PRP treatment in disc regeneration in animal models is a prerequisite for human clinical trials, we performed this meta-analysis to evaluate the efficacy of PRP treatment in retarding disc degeneration. This meta-analysis focused on the following outcomes: alterations in disc height, NP hydration as indicated by MRI T2 signal intensity, histological disc degeneration grade, and collagen II expression.

## 2. Materials and Methods

### 2.1. Statement of Our Reporting Standard

This meta-analysis complies with the Preferred Reporting Items for Systematic Reviews and Meta-Analyses (PRISMA) statement [43].

### 2.2. Search Strategy

A comprehensive literature search was performed using the following databases: MEDLINE, PubMed, EMBASE, the Cochrane Library, and the China National Knowledge Internet (CNKI). The last search date was October 16, 2016. English and Chinese papers were included in the present study. The search terms were “platelet-rich plasma/PRP” and “intervertebral disc/intervertebral disk”. To identify other potentially relevant studies, authors' bibliographies and the reference lists of each identified study were manually retrieved.

### 2.3. Inclusion and Exclusion Criteria

First, the title and abstract of each article were evaluated to eliminate irrelevant or reduplicative literature. The full text of the remaining papers was then screened in accordance with our inclusion and exclusion criteria. In this meta-analysis, we included preclinical controlled trials (randomized (RCT) or nonrandomized (N-RCT)) that investigated the efficacy of PRP treatment in disc regeneration. We focused on the following outcomes at the final follow-up time point: disc height alteration, MRI T2 signal intensity, histological disc degeneration grade, and collagen II expression. Any type of disc degeneration model, induced by needle puncture, mechanical loading, or chemonucleolysis was included regardless of the animal species. Similarly, we did not limit the mode of intervention in the control group. We excluded studies without the original data (e.g., reviews, letters, or editorials) and studies without the outcomes of interest.

### 2.4. Data Extraction

Data extraction from the eligible studies was performed by two independent reviewers. Disagreements between the reviewers were resolved after discussion, and consensus was eventually reached on all extracted information. The extracted information included the first author's name, publication year, animal species, disc degeneration model (traumatic or chemical assault), interventions in the control group, and the outcomes of interest. Additionally, when the outcomes of interest were analyzed at more than one time point, the data from the final time point was used. When a study contained multiple experimental groups, the PRP treatment group and the related control group were selected. MRI T2 signal intensity was classified using the Pfirrmann classification, with grades I to V being scored from 1 to 5 [44]. MRI T2 signal intensity determined using the Thompson classification was reclassified using the Pfirrmann classification by two reviewers. Histological degeneration grade was classified using the grading scale described by Nomura et al. [45], with grades 0 to 5 being scored from 0 to 5.

### 2.5. Quality Assessment

The methodological quality of the eligible studies was assessed according to the Collaborative Approach to Meta-Analysis and Review of Animal Data from Experimental Studies (CAMARADES) checklist [46]. A 9-point item list (each item received 1 point) was used to evaluate the risk of bias: (1) publication in a peer-reviewed journal; (2) statement describing animal temperature control; (3) randomization of treatment or control assignment; (4) allocation concealment; (5) blinded assessment of outcome; (6) avoidance of animal anesthetics with marked intrinsic properties; (7) sample size calculation; (8) statement of compliance with regulatory requirements; and (9) statement of possible conflict of interest.

### 2.6. Data Synthesis

We used Stata/SE 12.0 (Stata Corporation, College Station, TX, USA) to analyze the outcome data. The primary outcome was expressed as the standardized mean difference (SMD) and related 95% confidence interval (CI). We then combined each outcome of interest across the included studies using the random-effects model. Heterogeneity was examined using Cochrane's *Q*-test (*p* > 0.10 indicates significant heterogeneity) and the *I*^2^ metric (*I*^2^ < 50% indicates acceptable heterogeneity). Additionally, a subgroup analysis based on animal species, study design, disc degeneration model, or follow-up period was also performed to investigate the possible sources of heterogeneity in our results.

### 2.7. Sensitivity Analysis and Publication Bias Analysis

To evaluate the stability of the outcomes of the meta-analysis, a sensitivity analysis was performed. We also estimated the heterogeneity before and after omitting studies with obvious variance to identify potential sources of heterogeneity. In addition, a funnel plot was used to qualitatively assess the publication bias, and the Begg test and the Egger test were used to quantitatively assess the publication bias.

## 3. Results

### 3.1. Study Selection and Study Characteristics

**Figure 1:**
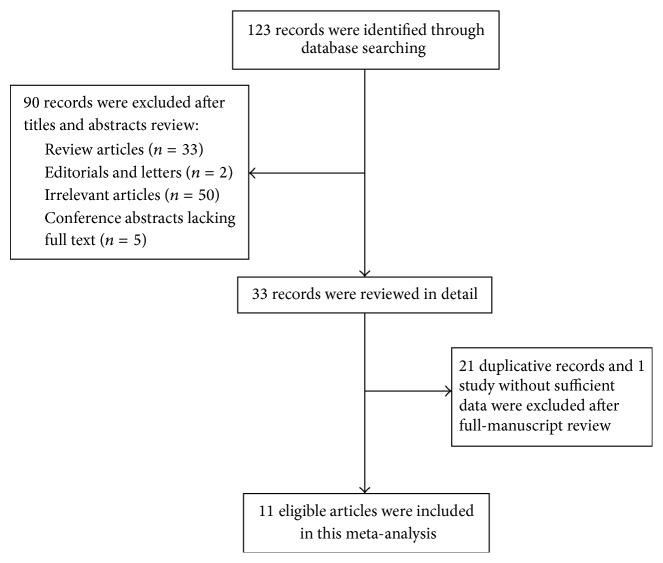
Flow diagram of the literature search and exclusion and inclusion process in the present meta-analysis.

The general process of literature retrieval and selection is shown in Figure 1. Briefly, the database searches yielded 123 literatures. After title and abstract screening, 90 records that were review articles, editorials, letters, irrelevant articles, or conference abstracts were excluded. Thereafter, 21 duplicate studies and 1 study without sufficient data were excluded following full-manuscript review. Finally, 11 studies were included in the present meta-analysis. Overall (as seen in Table 1), 419 discs were analyzed, of which 210 were treated with PRP and 209 were used as controls without PRP treatment. The animal species in the eligible studies included rabbit (9 studies, 81.82%), pig (1 study, 9.09%), and rat (1 study, 9.09%). Disc degeneration models in the eligible studies included needle puncture (8 studies, 72.73%), nucleus pulposus aspiration (2 studies, 18.18%), and chymopapain induction (1 study, 9.09%). Study designs included RCT (7 studies 63.64%) and N-RCT (4 studies, 36.36%). The median final follow-up period was 8 weeks (range: 2–12 weeks).

### 3.2. Literature Quality Assessment

Most of the studies in this meta-analysis did not perform a blinded assessment of the outcome and none of the studies included allocation concealment, a statement of animal temperature control, or sample size calculations. The quality assessment scores of the eligible studies ranged from 3 to 5. Among the 11 eligible studies, 3 scored 5 points, 4 scored 4 points, and 4 scored 3 points. The mean quality score was 3.9. The quality assessment of the eligible studies is summarized in Supplementary Table  1 (in Supplementary Material available online at https://doi.org/10.1155/2017/7919201).

### 3.3. Outcomes

As shown in Table 2, 7 studies evaluated the effects of PRP treatment on disc height. We found that disc height in the PRP-treated group was significantly higher than in the control group (SMD = 2.66, 95% CI: 1.86, 3.47, *p* = 0.000, *I*^2^ = 64.3%). Five studies reported the effects of PRP treatment on MRI T2 signal intensity. The Pfirrmann classification score in the PRP treatment group was significantly lower than in the control group (SMD = −3.29, 95% CI: −4.44, −2.13, *p* = 0.000, *I*^2^ = 68.9%). In 3 studies, the histological degeneration grade was significantly lower in the PRP treatment than in the control group (SMD = −4.28, 95% CI: −5.26, −3.30, *p* = 0.000, *I*^2^ = 0.0%). However, no difference in the expression of collagen II between the PRP treatment group and the control group was found in the 2 eligible studies (SMD = 25389.74, 95% CI: −27585.72, 78365.21, *p* = 0.348, *I*^2^ = 93.7%) (the Forests plots were shown in Supplementary Figure  1).

### 3.4. Subgroup Analysis

Because heterogeneity was detected in the effects of PRP treatment on disc degeneration, a subgroup analysis of disc height and MRI T2 signal intensity by animal species, study design, disc degeneration model, follow-up period, and disc height measurement was performed to investigate the potential source of heterogeneity. Due to the limited eligible studies and low heterogeneity of the histological degeneration grade outcome, we did not perform a subgroup analysis for collagen II expression or histological degeneration grade. Overall, no obvious differences were found between our main analysis and the subgroup analysis (Tables 3 and 4).

### 3.5. Sensitivity Analysis and Publication Bias

**Figure 2:**
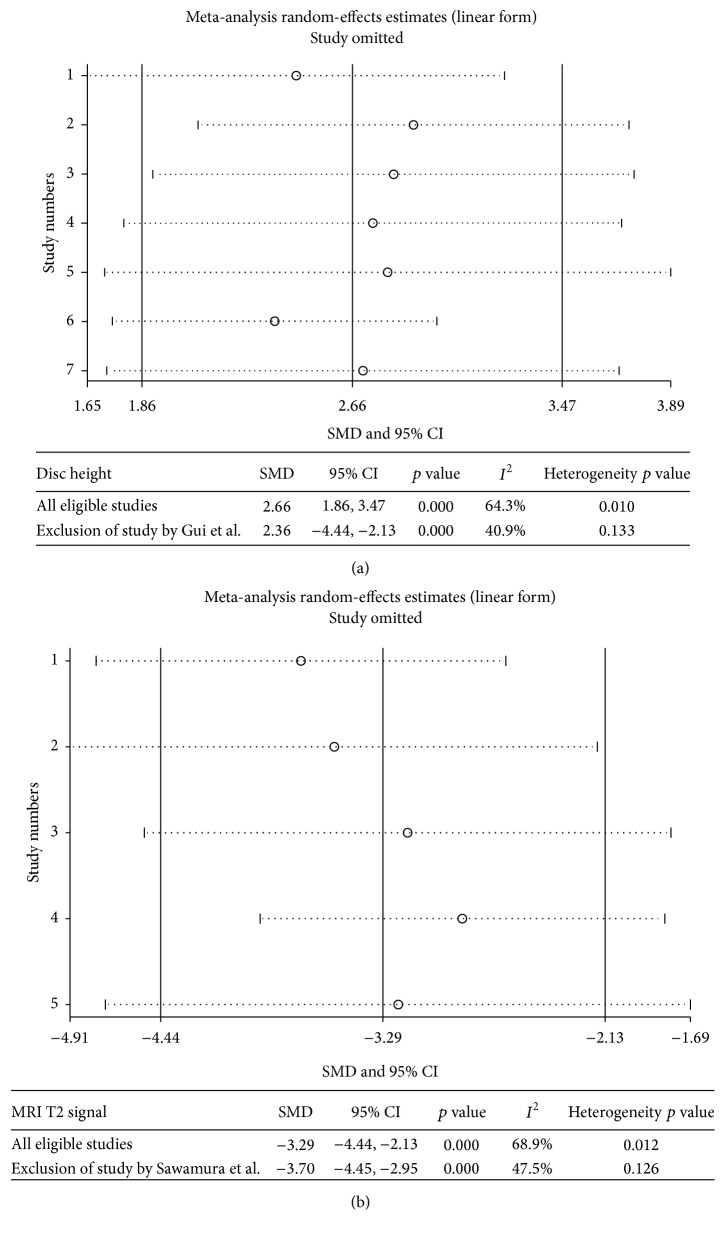
Sensitivity analysis for disc height (a) and MRI T2 signal intensity (b). The three-line table (a and b) indicates the SMD, 95% CI, and heterogeneity before and after the study with obvious variance was removed (SMD: standardized mean difference; 95% CI: 95% confidence interval).

In the sensitivity analysis for disc height outcome (Figure 2(a)), the initial heterogeneity (*I*^2^ = 64.3%, heterogeneity *p* value = 0.010) significantly decreased (*I*^2^ = 40.9%, heterogeneity *p* value = 0.133) when the study with obvious variance was removed (Gui et al. 2015, written in Chinese [41]). For the MRI T2 signal intensity outcome (Figure 2(b)), the initial heterogeneity (*I*^2^ = 68.9%, heterogeneity *p* value = 0.012) decreased when the study by Sawamura et al. [26] was removed (*I*^2^ = 47.5%, heterogeneity *p* value = 0.126). However, removal of this study did not dramatically alter the results of the overall pooled data for the effects of PRP treatment on disc height and MRI T2 signal intensity.

**Figure 3:**
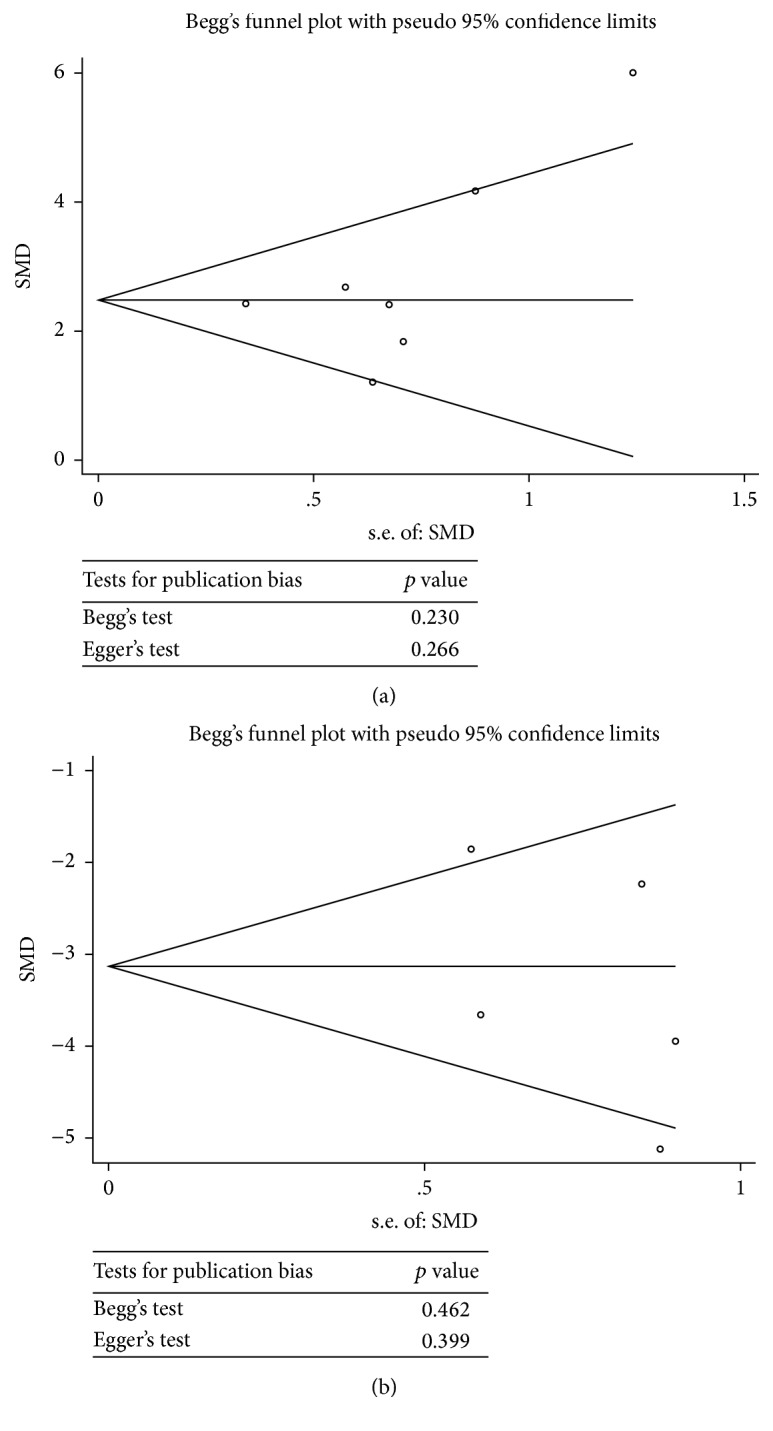
Funnel plot for the publication bias of studies on the effects of platelet-rich plasma (PRP) treatment on disc height (a) and MRI T2 signal intensity (b). The three-line table (a and b) indicates the *p* value for the Begg test and the Egger test, respectively.

No obvious asymmetry in the outcomes of disc height and MRI T2 signal intensity was observed on the funnel plot by visual inspection (Figure 3). The Egger test (*p* = 0.266 and *p* = 0.399 for disc height and MRI T2 signal, resp.) and the Begg test (*p* = 0.230 and *p* = 0.462 for disc height and MRI T2 signal, resp.) did not indicate any obvious publication bias. Because of the relatively limited number of studies on histological degeneration grade and collagen II expression outcomes, the publication bias analysis was not performed.

## 4. Discussion

PRP is a centrifuged blood product with high platelet content. Because it also contains a high level of growth factors, including TGF-*β*1, IGF, and PDGF, PRP has been used to promote bone formation and soft-tissue repair in clinical practice [47, 48]. Disc degeneration is regarded as a type of degenerative disease that is difficult to self-repair. Previously, several animal trials [26, 29, 33–42] have demonstrated that PRP treatment could inhibit the pathological process of intervertebral disc degeneration. However, the experimental parameters, including sample size, disc degeneration model induction method, follow-up period, and disc regeneration evaluation parameters, vary across studies. A quantitative analysis of synthesized homogeneous data may be helpful to overcome these shortcomings and to reach relatively reasonable conclusions. Therefore, we performed the present meta-analysis on the efficacy of PRP treatment in retarding disc degeneration in animal.

A degenerated disc undergoes serial pathological changes in its metabolism, biomechanics, geometric appearance, and cellular phenotype. These changes are characterized as reduced cell density, increased cell senescence and apoptosis, unbalanced matrix metabolism, upregulated inflammatory cytokines, and decreased disc height and NP hydration [10–12, 49]. In this meta-analysis, we mainly investigated the effects of PRP treatment on four important disc regeneration parameters: alteration of disc height, MRI T2 signal intensity, histological degeneration grade, and collagen II expression. Notably, our main analysis demonstrated that PRP treatment could significantly increase disc height and MRI T2 signal intensity while reducing the histological degeneration grade (all *p* value < 0.05, Table 2). Consistent with this finding, the individual in vivo [26, 29, 33–42] and in vitro studies [28–32] also demonstrated that PRP treatment has protective effects on the disc, such as promoting cell proliferation and matrix synthesis, decreasing cell apoptosis, increasing disc hydration, and increasing disc height. Together, our meta-analysis and these studies suggest that PRP treatment may be a promising therapy for retarding disc regeneration.

Because heterogeneity in disc height (*I*^2^ = 64.3%) and MRI T2 image (*I*^2^ = 68.9%) was observed in our main meta-analysis, a subgroup analysis to determine significantly sources of heterogeneity was performed. We did not conduct a subgroup analysis of the histological degeneration grade and collagen II expression due to the undetected heterogeneity (*I*^2^ = 0.0%) and limited number of eligible studies (*n* = 2), respectively. Generally, the subgroup analysis results were consistent with those of the main analysis. However, the subgroup analysis of disc height outcome showed no significant difference in the disc height index in pig [29] or in the chymopapain-induced degeneration model after PRP treatment (all *p* value = 0.058, Table 3), which contradicts the significant difference found in the main analysis of disc height (*p* = 0.000). This indicates that the animal species and degeneration model induction method may be potential sources of heterogeneity for disc height outcome. Similarly, subgroup analysis of MRI T2 signal intensity indicated that follow-up period may be a potential source of heterogeneity for the T2 weighted MRI image. Based on these findings, it is possible that PRP treatment in large animals or in an animal degeneration model with heavy damage or with a short follow-up period does not exhibit statistically significant disc regeneration efficacy.

Sensitivity analysis is a common method for investigating study stability. Because the number of eligible studies for the histological degeneration grade and collagen II expression outcomes was low, we only performed the sensitivity analysis for studies that evaluated the efficacy of PRP treatment with respect to disc height restoration and TMRI T2 signal intensity. Our results showed that the heterogeneity in the disc height and TMRI T2 signal intensity outcomes decreased significantly when the study by Gui et al. [41] and the study by Sawamura et al. [26] were omitted (from 64.3% to 40.9% and from 68.9% to 47.5%, resp.). However, this did not dramatically alter the overall result of the pooled data with respect to changes in disc height and TMRI T2 signal intensity after PRP treatment. Moreover, no significant publication bias was detected for the disc height and MRI T2 signal intensity outcomes. These results imply that our meta-analysis results were stable. Additionally, these results indicate that certain underlying factors in the abovementioned studies may be a potential source for the heterogeneity of the synthesized data for changes in disc height and MRI T2 signal intensity. After screening the details of the 11 eligible studies, we speculate that the short follow-up period (2 weeks in the study by Gui et al. [41]) and the more exacerbated disc degeneration status (induction by NP aspiration in the study by Sawamura et al. [26]) are possible contributors to the decrease in heterogeneity when the corresponding studies were omitted.

This meta-analysis has several limitations. First, the accessible data of some of the eligible studies were relatively limited and not similar enough, which directly affected the cogency of our results. Second, because of our search strategy focused on English and Chinese articles, publication bias and selection bias were inevitable. Third, sensitivity analysis and publication bias analysis were not performed on the histological degeneration grade and collagen II expression outcomes due to the insufficient number of eligible studies. Fourth, when it comes to the molecular and cellular responses to regeneration, there are no objective criteria that truly identify intervertebral disc regeneration at the structural level. Because of these limitations, a more comprehensive literature-election process and an updated meta-analysis on the present topic that includes a greater number of eligible studies should be considered in the future.

## 5. Conclusions

This meta-analysis study demonstrated that PRP treatment plays an important role in disc regeneration, especially in restoring disc height of rodent rabbit and rat, reducing histological degeneration grade, and increasing MRI T2 signal intensity. This study provides direct theoretical support for the future application of PRP in retarding human disc degeneration.

## Supplementary Material

Supplementary Table 1. Quality assessment of the included studies (NA: Not available). Supplementary Figure 1. Forest plots for the main analysis of disc height (A), T2 weighted MRI (B), histological degeneration grade (C) and collagen II expression (D).

## Figures and Tables

**Table 1:** Characteristics of the included studies.

First author	Publication year	Country	Manuscript language	Animal species	Disc degeneration model	Study design	Observation time (weeks)	PRP description
Nagae et al. [36]	2007	Japan	English	Rabbit	Nucleus pulposus aspiration	N-RCT	8	Autologous
Sawamura et al. [26]	2009	Japan	English	Rabbit	Nucleus pulposus aspiration	N-RCT	8	Autologous
Chen et al. [29]	2009	China	English	Pig	Chymopapain induction	N-RCT	8	Allogenic
Gullung et al. [34]	2011	USA	English	Rat	Needle puncture	RCT	4	Not available
Hu et al. [40]	2012	China	Chinese	Rabbit	Needle puncture	RCT	2	Autologous
Obata et al. [37]	2012	Japan	English	Rabbit	Needle puncture	N-RCT	8	Autologous
Meng et al. [42]	2013	China	Chinese	Rabbit	Needle puncture	RCT	8	Allogenic
Gui et al. [41]	2015	China	Chinese	Rabbit	Needle puncture	RCT	2	Autologous
Gui et al. [33]	2015	China	English	Rabbit	Needle puncture	RCT	6	Autologous
Wang et al. [38]	2016	China	English	Rabbit	Needle puncture	RCT	8	Allogenic
Yang et al. [39]	2016	China	English	Rabbit	Needle puncture	RCT	12	Autologous

RCT: randomized controlled trial; N-RCT: nonrandomized controlled trial.

**Table 2:** Efficacy of PRP injection in retarding intervertebral disc degeneration.

Outcomes	Number of studies	SMD	95% CI	*p* value	*I* ^2^	Heterogeneity *p* value
Disc height	7	2.66	1.86, 3.47	0.000	64.3%	0.010
T2 weighted MRI	5	−3.29	−4.44, −2.13	0.000	68.9%	0.012
Histological degeneration grade	3	−4.28	−5.26, −3.30	0.000	0.0%	0.419
Collagen II expression	2	25389.74	−27585.72, 78365.21	0.348	93.7%	0.000

SMD: standardized mean difference; 95% CI: 95% confidence interval.

**Table 3:** Subgroup analysis of the disc height based on animal species, study design, disc degeneration model, follow-up period, and disc height measurement.

Subgroup analysis index	Number of studies	SMD	95% CI	*p* value	*I* ^2^
*Animal species*					
Rabbit	5	3.14	2.19, 4.08	0.000	62.8%
Pig	1	1.21	−0.04, 2.45	0.058	NA
Rat	1	1.83	0.44, 3.22	0.010	NA
*Study design*					
N-RCT	3	2.50	0.91, 4.09	0.002	73.5%
RCT	4	2.82	1.75, 3.89	0.000	66.6%
*Disc degeneration model*					
Nucleus pulposus aspiration	1	4.17	2.46, 5.89	0.000	NA
Chymopapain induction	1	1.21	−0.04, 2.45	0.058	NA
Needle puncture	5	2.68	1.86, 3.51	0.000	55.7%
*Follow-up period*					
2 weeks	1	6.01	3.58, 8.44	0.000	NA
4 weeks	1	1.83	0.44, 3.22	0.010	NA
6 weeks	1	2.67	1.55, 3.80	0.000	NA
8 weeks	4	2.43	1.49, 3.37	0.000	60.5%
*Disc height measurement *					
Absolute disc height	2	2.95	0.66, 5.23	0.012	67.2%
DHI%	5	2.59	1.65, 3.53	0.000	64.3%

RCT: randomized controlled trial; N-RCT: nonrandomized controlled trial. SMD: standardized mean difference; 95% CI: 95% confidence interval. DHI: disc height index.

**Table 4:** Subgroup analysis of T2 weighted MRI image based on animal species, study design, disc degeneration model, and follow-up period.

Subgroup analysis index	Number of studies	SMD	95% CI	*p* value	*I* ^2^
*Animal species*					
Rabbit	5	−3.29	−4.44, −2.13	0.000	68.9%
*Study design*					
N-RCT	1	−1.85	−2.98, −0.73	0.001	NA
RCT	4	−3.70	−4.45, −2.95	0.000	47.5%
*Disc degeneration model *					
Nucleus pulposus aspiration	1	−1.85	−2.98, −0.73	0.001	NA
Needle puncture	4	−3.70	−4.45, −2.95	0.000	93.5%
*Follow-up period *					
2 weeks	2	−3.04	−4.24, −1.83	0.784	96.0%
6 weeks	1	−5.12	−6.83, −3.41	0.000	NA
8 weeks	1	−1.85	−2.98, −0.73	0.001	NA
12 weeks	1	−3.65	−4.81, −2.50	0.000	NA

RCT: randomized controlled trial; N-RCT: nonrandomized controlled trial. SMD: standardized mean difference; 95% CI: 95% confidence interval.
