# Supplementary material for: Efficacy of Platelet-Rich Plasma in Retarding Intervertebral Disc Degeneration: A Meta-Analysis of Animal Studies
Source: Biomed Res Int. 2017 Jul 2;2017:7919201. doi: 10.1155/2017/7919201 (PMC5511641; doi:10.1155/2017/7919201)

Supplementary Table 1. Quality assessment of the included studies (NA: Not available).

Author	Publication in a peer-reviewed journal	Animal temperature control	Randomization of treatment or control assignment	Allocation concealment	Blinded assessment of outcome	Animal anesthetics without marked intrinsic properties	Sample size calculation	Compliance with regulatory requirements	Statement of possible conflict of interest	Total score
Nagae et al.	1	NA	NA	NA	NA	1	NA	1	NA	3
Sawamura et al.	1	NA	NA	NA	NA	1	NA	1	NA	3
Chen et al.	1	NA	NA	NA	NA	1	NA	1	1	3
Gullung et al.	1	NA	1	NA	1	1	NA	1	NA	5
Hu et al.	1	NA	1	NA	NA	1	NA	1	NA	5
Obata et al.	1	NA	NA	NA	NA	1	NA	1	NA	3
Meng et al.	1	NA	1	NA	1	1	NA	1	NA	5
Gui et al.	1	NA	1	NA	NA	1	NA	1	NA	4
Gui et al.	1	NA	1	NA	NA	1	NA	1	NA	4
Wang et al.	1	NA	1	NA	NA	1	NA	1	NA	4
Yang et al.	1	NA	1	NA	NA	1	NA	1	NA	4

Forest plots for the main analysis of disc height (A), T2 weighted MRI (B), histological degeneration grade (C) and collagen II expression (D.)

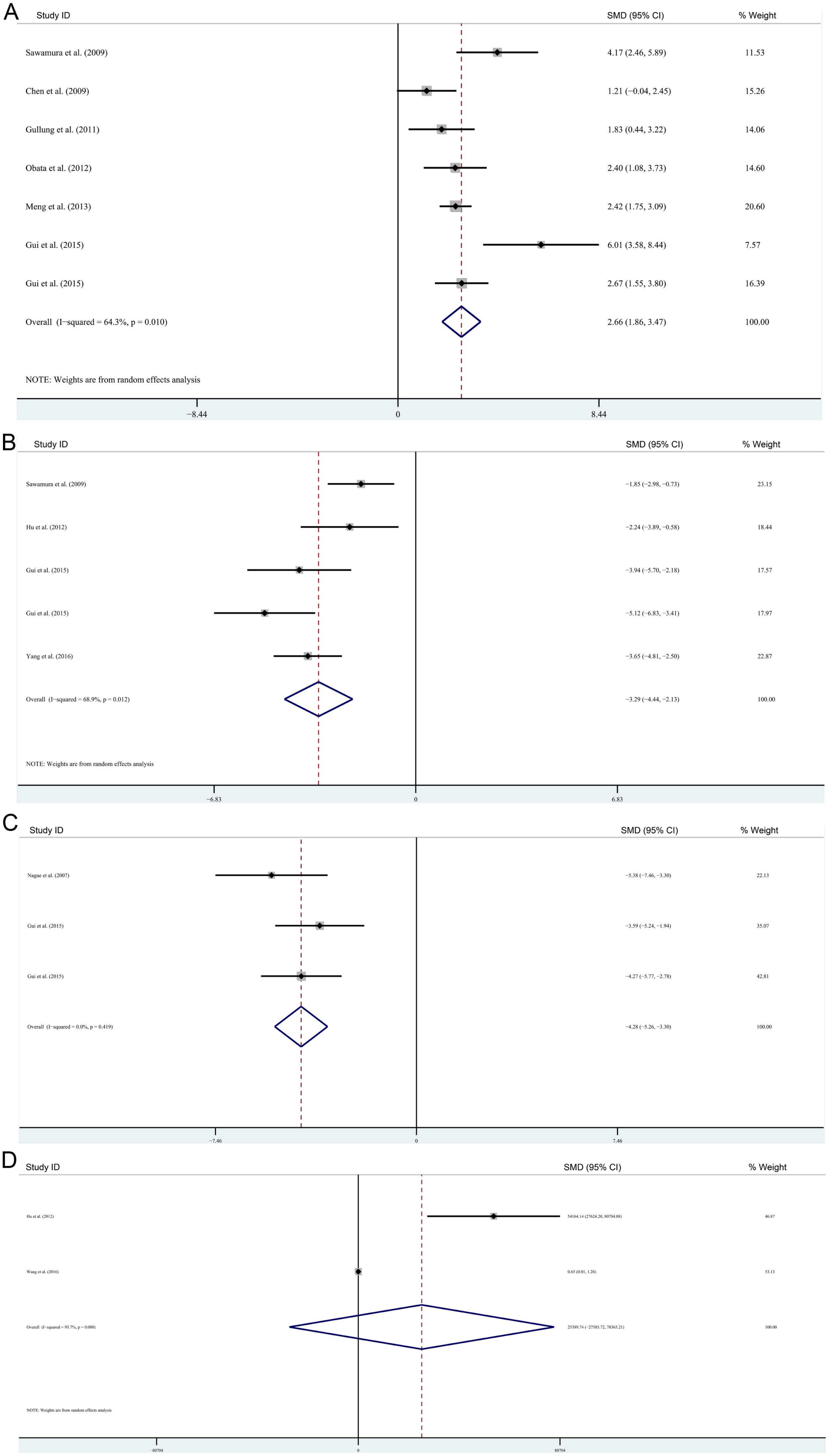

Supplement: Supplementary file 1 — Supplementary Table 1. Quality assessment of the included studies (NA: Not available). Supplementary Figure 1. Forest plots for the main analysis of disc height (A), T2 weighted MRI (B), histological degeneration grade (C) and collagen II expression (D). [file 7919201.f1.pdf]
